# Maximal COX-2 and ppRb expression in neurons occurs during early Braak stages prior to the maximal activation of astrocytes and microglia in Alzheimer's disease

**DOI:** 10.1186/1742-2094-2-27

**Published:** 2005-11-21

**Authors:** Jeroen JM Hoozemans, Elise S van Haastert, Robert Veerhuis, Thomas Arendt, Wiep Scheper, Piet Eikelenboom, Annemieke JM Rozemuller

**Affiliations:** 1 Department of Neuropathology, Academic Medical Center, P.O. Box 22700, 1100 DE Amsterdam, The Netherlands; 2 Neurogenetics Laboratory, Academic Medical Center, University of Amsterdam, Amsterdam, The Netherlands; 3 Department of Psychiatry, VU University medical center, Amsterdam, The Netherlands; 4 Department of Clinical Chemistry and Alzheimer Center, VU University medical center, Amsterdam, The Netherlands; 5 Department of Neuroanatomy, Paul Flechsig Institute for Brain Research, University of Leipzig, Leipzig, Germany

**Keywords:** Alzheimer's disease, astrocytes, cell cycle, cyclooxygenase-2, microglia, retinoblastoma protein

## Abstract

Neuronal expression of cyclooxygenase-2 (COX-2) and cell cycle proteins is suggested to contribute to neurodegeneration during Alzheimer's disease (AD). The stimulus that induces COX-2 and cell cycle protein expression in AD is still elusive. Activated glia cells are shown to secrete substances that can induce expression of COX-2 and cell cycle proteins *in vitro*. Using *post mortem *brain tissue we have investigated whether activation of microglia and astrocytes in AD brain can be correlated with the expression of COX-2 and phosphorylated retinoblastoma protein (ppRb). The highest levels of neuronal COX-2 and ppRb immunoreactivity are observed in the first stages of AD pathology (Braak 0–II, Braak A). No significant difference in COX-2 or ppRb neuronal immunoreactivity is observed between Braak stage 0 and later Braak stages for neurofibrillary changes or amyloid plaques. The mean number of COX-2 or ppRb immunoreactive neurons is significantly decreased in Braak stage C compared to Braak stage A for amyloid deposits. Immunoreactivity for glial markers KP1, CR3/43 and GFAP appears in the later Braak stages and is significantly increased in Braak stage V-VI compared to Braak stage 0 for neurofibrillary changes. In addition, a significant negative correlation is observed between the presence of KP1, CR3/43 and GFAP immunoreactivity and the presence of neuronal immunoreactivity for COX-2 and ppRb. These data show that maximal COX-2 and ppRb immunoreactivity in neurons occurs during early Braak stages prior to the maximal activation of astrocytes and microglia. In contrast to *in vitro *studies, *post mortem *data do not support a causal relation between the activation of microglia and astrocytes and the expression of neuronal COX-2 and ppRb in the pathological cascade of AD.

## Findings

Aberrant expression of cyclins, cyclin dependent kinases (CDKs) and their inhibitors has been observed in *post mitotic *neurons in Alzheimer's disease (AD) [1,2]. Proteins that normally function to control cell cycle progression in actively dividing cells may play a role in the death of *post mitotic *neurons in AD [3]. The retinoblastoma protein (pRb) regulates cell proliferation by controlling progression through the restriction point within the G1-phase of the cell cycle [4]. pRb sequesters members of the E2F gene family of transcription factors. Cell cycle-dependent phosphorylation of pRb by CDKs inactivates pRb and inhibits pRb target binding, allowing cell cycle progression. The expression of phosphorylated pRb (ppRb) immunoreactivity in AD neurons has previously been described [5,6]. In the midfrontal and temporal cortex ppRb immunoreactivity can be most prominently detected in the nucleus of the large pyramidal neurons of layers III and V, and is rarely detected in neurofibrillary tangles. Recent studies have shown that neuronal cyclooxygenase-2 (COX-2) expression in AD parallels the expression of cell cycle proteins in neurons [6-8]. Previously, we observed colocalization of COX-2 with ppRb in neurons in the temporal cortex of AD and control cases [6]. Increased neuronal COX-2 expression leads to increased expression of cell cycle mediators in *post mitotic *neurons, as shown using a transgenic mouse model with increased neuronal COX-2 expression [9].

Once activated, microglia and astrocytes are capable of producing a variety of pro-inflammatory mediators and potentially neurotoxic substances [10], of which some have been shown to potentially induce COX-2 and cell cycle protein expression *in vitro *[3,11-13]. It has been shown that interleukin-1β induces COX-2 expression in neuronal cell models [11,12]. and conditioned medium from β amyloid (Aβ) peptide stimulated microglia induces expression of cell cycle proteins in neurons followed by cell death [13]. These *in vitro *findings indicate that the activation of microglia may play an important role in the expression of COX-2 and cell cycle proteins in neurons. *Post mortem *as well as *in vivo *studies indicate that microglial activation already occurs at an early stage in AD pathology [14,15]. Cell cycle changes and increased neuronal COX-2 expression have also been shown to be early events in AD [1,7,16,17]. We therefore hypothesized that neuronal expression of COX-2 and ppRb would be associated with increased presence and activation of glial cells.

**Figure 1:**
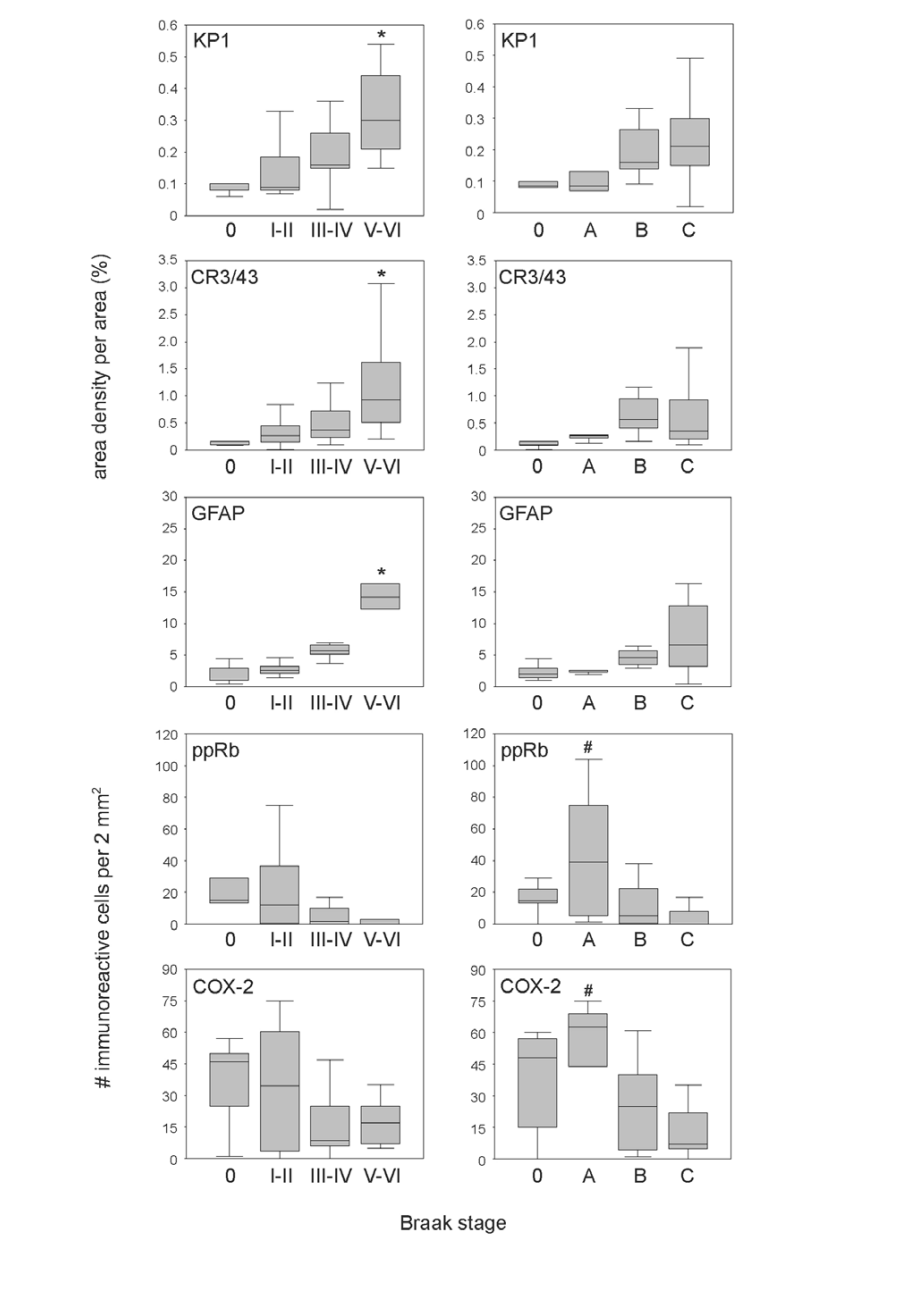
**Immunoreactivity scores for KP1, CR3/43, GFAP, ppRb and COX-2 in the temporal cortex of nondemented control and AD cases**. Immunohistochemical stainings were performed as described previously [6]. The following primary antibodies were used: rabbit polyclonal anti-COX-2 (Cayman, Ann Arbor, MI), rabbit anti-phosphoserine pRb (pSer 795, Cell Signaling, Beverly, MA). Mouse anti-CD68 (KP1) and mouse anti-HLA-DP, DQ, DR (CR3/43) were obtained from DAKO (Heverlee, Belgium). Mouse anti-Glial Fibrillary Acidic Protein (GFAP) was obtained from Monosan (clone 6F2, Uden, The Netherlands). Morphometric investigation was aimed at determining the area density occupied by the immunoreactive glial cells in the cortical layer. The area density (%) was quantified using Image-Pro Plus analysis software (Media Cybernetics, Silver Spring, MD). Immunoreactive neurons (COX-2 and ppRb) were counted in a total area of 2 mm^2^. Neurons were distinguished from non-neuronal cells by nuclear size and shape. Values of cases are grouped according to the Braak stage for neurofibrillary changes (O, I-II, III-IV, V-VI) or Aβ deposits (O, A, B, C). Results are expressed as box plots. The box represents the interquartile range that contains 50% of the values. The whiskers extend from the box to the highest and lowest values. The line across the box indicates the median. Kruskall-Wallis test was used to evaluate differences between groups followed by the Mann-Whitney U test, to test differences between pairs of groups. Correlation analysis was done using the Pearson parametric and Spearman non-parametric method. * p < 0.05 versus Braak stage O. # p < 0.05 versus Braak stage C.

Using *post mortem *brain tissue we have investigated whether activation/occurrence of microglia and astrocytes in AD brain can be correlated with the neuronal expression of COX-2 and ppRb during AD pathogenesis. Staging of AD was neuropathologicallly evaluated according to Braak and Braak [18]. Demographic characteristics of the cases used in this study are shown in table 1. For each case the area density of the immunoreactivity for KP1, CR3/43 and GFAP in the mid-temporal cortex was determined. KP1 (anti-CD68) is a marker for phagocytic microglia (and macrophages) and CR3/43 detects the class II antigens HLA-DP, DQ, DR and is generally used as a marker for activated microglia. GFAP (Glial Fibrillary Acidic Protein) is strongly and specifically expressed in astrocytes. Group summaries are expressed as box-plots for each Braak stage for neurofibrillary changes or amyloid deposits [18] (figure 1). All three markers show a gradual increase with increasing pathology. Correlation analysis reveals a statistically significant (p < 0.05) positive correlation between the Braak scores for neurofibrillary changes (NF) or Aβ deposits (AMY) and immunoreactivity for KP1 (NF, 0.671; AMY, 0.432), CR3/43 (NF, 0.564; AMY, 0.323), and GFAP (NF, 0.690; AMY, 0.424). A statistically significant increase was observed in Braak stage V-VI for KP1 (p = 0.001), CR3/43 (p = 0.008), and GFAP (p < 0.001) compared to Braak stage 0. Neuronal ppRb and COX-2 immunoreactivity are expressed as number of immunoreactive neurons per 2 mm^2 ^(figure 1). A significant (p < 0.05) negative correlation was observed between the Braak score for neurofibrillary changes and ppRb (-0.414) or COX-2 (-0.346), and between the Braak score for Aβ plaques and COX-2 (-0.537).

**Table 1:** Demographic characteristics of the cases used in this study. Shown are differences between groups of the cases used in this study. [PMI post-mortem interval, SD standard deviation].

	**Braak score for neurofibrillary changes**				
	
	O	I–II	III–IV	V–VI	
	
**n**	5	16	10	9	
**male/female**	3/2	6/10	0/10	3/6	
**mean age ± SD (years)**	62 ± 10	83 ± 8	89 ± 4	76 ± 7	
**PMI ± SD (hrs:min)**	8:00 ± 4:30	7:30 ± 2:30	6:30 ± 2:30	5:00 ± 1:30	
	**Braak score for amyloid deposits**				
		
	O	A	B	C	total
	
**n**	7	6	11	16	40
**male/female**	4/3	3/3	3/8	2/14	12/28
**mean age ± SD (years)**	69 ± 12	79 ± 4	85 ± 10	82 ± 10	80 ± 11
**PMI ± SD (hrs:min)**	7:00 ± 4:00	8:30 ± 3:00	7:00 ± 2:30	6:00 ± 2:00	6:30 ± 2:30

Although it is tempting to assume that these stages reflect the clinical changes, this study aims to show the relation between different molecular pathologically defined events. Cases with Braak stage A used in this study had either Braak stage I or II for neurofibrillary changes. In Braak stage A for amyloid low densities of amyloid plaques are only found in the temporal cortex and other parts of the isocortex [18]. Activated glial cells are mostly associated with neuritic plaques not with diffuse Aβ plaques [10]. This is in agreement with our data which shows a gradual increase in microglia and astrocytes with the Braak score for neurofibrillary changes and high levels of activated glial cells in cases with Braak score B and C (figure 1).

We observed maximal neuronal ppRb and COX-2 immunoreactivity in Braak stages 0 and A. No significant difference in ppRb and COX-2 immunoreactivity was observed between the Braak stages for neurofibrillary changes. The maximal ppRb and COX-2 immunoreactivity in stage A did not significantly differ from stage O. However, we did observe a significant decrease in Braak stage C compared to stage A. These findings contradict previous studies that have shown increased neuronal COX-2 expression [19,20] and ppRb immunoreactivity in AD cases [5]. In the present study the patients are grouped according to the Braak stage instead of being defined as control or AD. Other, previously described [17], discrepancies are most likely due to differences in pathological disease state and investigated brain area, methods of analysis, as well as technical issues. The data presented in this study are in agreement with the findings of Yermakova and O'Banion [17]. In an immunohistochemical study they found a decrease in the number of COX-2 immunoreactive neurons in advanced stages of AD. A similar trend, as shown in the present study, was observed in the hippocampus comparing the mean neuronal COX-2 immunoreactivity with the Braak score for NF. A non-significant higher mean level in Braak stage I-II was also reported [17]. The levels of neuronal COX-2 expression observed in *post mortem *brain tissue correlate well with recent clinical data presented by Combrinck and colleagues [21] describing, compared to control patients, higher prostaglandin E2 levels in the cerebrospinal fluid in patients with mild memory impairment, but lower in those with more advanced AD.

A significant negative correlation was observed between the area density of KP1 and the immunoreactivity for ppRb (-0.414, p = 0.007) and COX-2 (-0.366, p = 0.020). These data suggest no (positive) relation between neuronal expression of COX-2 or ppRb and the increased glial response observed during AD pathology. Although suggested by *in vitro *studies, our evaluation of *post mortem *brain tissue suggests that it is very unlikely that activation of microglia or astrocytes cause neuronal expression of COX-2 and ppRb in AD. Although the involvement of activated glia in the initial upregulation of these factors seems unlikely, we cannot exclude the involvement of glia in the regulation of COX-2 or cell cycle protein expression in neurons at later stages of pathology.

COX-2 and cell cycle changes can be detected in neurons that are vulnerable for developing neurodegenerative changes that are associated with AD [6,16,22]. This implies that COX-2 and neuronal cell cycle changes occur in the early steps of AD neurodegeneration. Moreover, high levels of neuronal COX-2, ppRb, cyclin D1 and cyclin E are found in the temporal cortex of cases which have diffuse Aβ deposits while fibrillar/neuritic plaques are absent [6,7]. Various i*n vitro *studies using neuronal models show that Aβ peptide induces COX-2 [20] and phosphorylation of pRb [23,24], which is followed by neuronal cell death. In this perspective, the current emerging data on the early role of oligomeric and protofibrilic forms of Aβ in AD is very interesting [25,26]. Whether COX-2 and cell cycle proteins are part of the molecular mechanisms involved in the response to intraneuronal accumulation of Aβ and the consequent impaired synaptic function needs to be addressed in future studies.

## Competing interests

The author(s) declare that they have no competing interests.

## Authors' contributions

JJMH participated in the design of the study, performed the statistical analysis and prepared the manuscript. ESvH carried out the immunohistochemical analyis and quantification of the immunohistochemical data. RV has been involved in the collection of the human post mortem brain material. TA, WS, PE and AJMR participated in the design of the study and helped to draft the manuscript. All authors read and approved the final manuscript.
